# Conditioning on a Collider May or May Not Explain the Relationship Between Lower Neuroticism and Premature Mortality in the Study by Gale et al. (2017): A Reply to Richardson, Davey Smith, and Munafò (2019)

**DOI:** 10.1177/0956797619833325

**Published:** 2019-02-22

**Authors:** Alexander Weiss, Catharine R. Gale, Iva Čukić, G. David Batty, Andrew M. McIntosh, Ian J. Deary

**Affiliations:** 1 Centre for Cognitive Ageing and Cognitive Epidemiology, Department of Psychology, University of Edinburgh; 2 Department of Psychology, School of Philosophy, Psychology & Language Sciences, University of Edinburgh; 3 MRC Lifecourse Epidemiology Unit, University of Southampton; 4 Department of Epidemiology & Public Health, University College London; 5 Division of Psychiatry, University of Edinburgh

We (Gale et al., 2017) analyzed data on 321,456 UK Biobank participants to address why higher neuroticism is sometimes related to lower mortality (e.g., Korten et al., 1999). We noted that the studies that revealed an inverse relationship between neuroticism and mortality included self-rated health as a predictor, perhaps because it is associated with mortality, even when multiple objective measures of health status are included in models (for reviews, see Benyamini & Idler, 1999; Idler & Benyamini, 1997). We therefore tested whether including self-rated health, which was modestly related to neuroticism, *r*_S_ = .23 (Gale et al., p. 1348), in a model changed the sign of the neuroticism–mortality relationship. We found that it did so and then set out to test two possible explanations for this phenomenon.

The first possible explanation that we tested was that self-rated health moderated the neuroticism–mortality association. We therefore included a Neuroticism × Self-Rated Health interaction in our models and also examined the association between neuroticism and mortality at each self-rated health stratum. The interaction was significant only for cancer death; analyses stratified by self-rated health suggested that neuroticism was protective against all-cause mortality among participants with “fair” self-rated health and protective against cancer mortality among participants with “fair” or “poor” self-rated health (Gale et al., 2017, pp. 1350–1352).

The second possible explanation that we tested was that self-rated health acted as a negative suppressor (Tzelgov & Henrik, 1991). In other words, we tested whether including self-rated health in models controlled for aspects of neuroticism related to poorer health. This explanation is based on the fact that in the presence of the pattern of correlations that gives rise to the statistical phenomenon known as negative suppression, mediation analyses will result in opposite signs for the direct and indirect effects (Tzelgov & Henrik, 1991); this is sometimes called “inconsistent mediation” (see MacKinnon, Krull, & Lockwood, 2000, p. 175). The self-rated-health and personality literatures, as much as it is possible for longitudinal and prospective studies to do so, provide findings that are consistent with this explanation. This literature shows that neuroticism has a negative effect on (is on a causal pathway to) self-rated health (Löckenhoff, Terracciano, Ferrucci, & Costa, 2012), that neuroticism has a positive effect on mortality (Graham et al., 2017), and that poor self-rated health has a positive effect on mortality (for reviews, see Benyamini & Idler, 1999; Idler & Benyamini, 1997), including even non-illness-related mortality (Heistaro, Jousilahti, Lahelma, Vartiainen, & Puska, 2001). Given this literature, it is plausible that neuroticism would have a direct and positive effect on mortality and an indirect negative effect (via self-rated health) on mortality. This was in fact found, although only in women, in a previous study (Ploubidis & Grundy, 2009).

Because personality–health associations are often limited to one or a few personality facets (e.g., Terracciano et al., 2009), we followed a reviewer’s suggestion and tested whether variance related to health-harming but not health-helping facets of neuroticism might have been controlled for by the inclusion of self-rated health in the model. Our analyses proceeded as follows. We first had to contend with the fact that participants completed the short Neuroticism scale from the Revised Eysenck Personality Questionnaire (EPQ-R; Eysenck, Eysenck, & Barrett, 1985), which does not operationalize facets. We therefore operationalized facets by conducting an exploratory bifactor analysis. This involves extracting factors using exploratory factor analysis and then rotating these factors so that all items have high loadings on a general factor and each item has a high loading on, at most, one of two or more special factors that are orthogonal to the general factor (Jennrich & Bentler, 2011, 2012). The latent variable for each special factor, therefore, is made up only of item variance related to that special factor; the latent variable score for the general factor consists only of the common item variance. A reviewer advised that we use bifactor analysis because, by not doing so, such as by using simple sum scores, it is not possible to determine whether an association between a facet and an outcome is related to the facet or the general factor, which would share a considerable portion of variance with the facet (see Wiernik, Wilmot, & Kostal, 2015, for a discussion). Alongside the general neuroticism factor, the analysis yielded two special factors, representing facets that we labeled “anxious/tense” and “worried/vulnerable” (see Fig. 1).

**Fig. 1.**
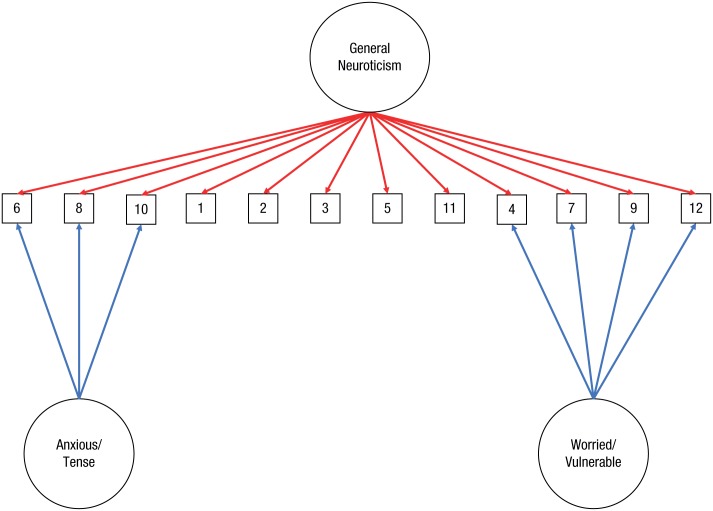
Bifactor structure of neuroticism in the UK Biobank data. The circle and arrows at the top of the figure represent loadings of the general factor onto the 12 Neuroticism items from the short form of the Revised Eysenck Personality Questionnaire (Eysenck, Eysenck, & Barrett, 1985). The circles and arrows at the bottom of the figure represent loadings of facets onto specific Neuroticism items from this scale. All loadings were positive. Loadings less than |.3| are not presented. Item numbers in the figure correspond to the position of the items presented in Table 9 in the article by Eysenck et al. Item wording is presented in Appendix 2 of Eysenck et al.

We then examined associations between these latent variable scores and each mortality outcome in one model that included age, sex, and the general neuroticism factor and a second model that additionally included all the covariates, including self-rated health. The critical analyses were those related to the first model (Gale et al., 2017, Table 4). There could be no conditioning on a collider in this model because neither self-rated health nor other health-related covariates were included. For death from all causes, cancer, cardiovascular disease, and respiratory disease, but not from external causes, higher worried/vulnerable scores were associated with reduced risk. Anxious/tense scores were not associated with any mortality outcomes. Only the relationship between the worried/vulnerable facet and all-cause mortality survived adjusting for the covariates *and* correction for the false discovery rate.

Richardson and colleagues (2019) proposed that our findings relating to conditioning on self-rated health could be spurious if neuroticism and mortality risk factors independently influenced self-rated health because, in these circumstances, self-rated health is a collider (note that in their model, self-rated health is a consequence and not a cause of poor health). To explore this possibility, they examined associations between neuroticism and the health-related covariates from our analyses, both in the total sample and stratified by self-rated health. For some covariates, they found that neuroticism was associated with increased risk in the total sample and reduced risk in self-rated health strata, an example of Simpson’s (1951) paradox. They interpreted this as evidence that our finding of a health-protective effect of neuroticism was spurious.

We thank Richardson and colleagues for their comments. Collider bias may lead to results such as those we reported. However, as noted, negative suppression may also lead to the same results, and our facet-level analyses that we described above did not include possible colliders, and so seems to support this explanation of the phenomenon. We also wish to be clear that we did not conclude from our previous study that we think the phenomenon in question was the result of neuroticism having a different effect at different levels of self-rated health, which seems to be what Richardson and colleagues surmised. To prevent further misunderstanding, we concluded in the final paragraph that “perhaps the most promising avenue for future research would be a closer examination of the role of Neuroticism’s facets” (Gale et al., 2017, p. 1355). In other words, we judged that our results supported the possibility that self-rated health acted as a negative suppressor that revealed the effects of a specific facet of neuroticism.

In response to their Commentary, we now report further analyses that we carried out to test whether their findings can also be explained by the worried/vulnerable facet.

## Method

To start, we tested whether neuroticism’s bifactor structure replicated in two independent data sets: 8,158 participants in Generation Scotland (Smith et al., 2006) and the 1,434 participants used to develop the EPQ-R (Eysenck et al., 1985). We then used data from the participants from our previous article and from Richardson and colleagues’ analyses to conduct three sets of new analyses, which we describe below.

First, we used multinomial logit regression to examine associations between self-rated health strata and the latent neuroticism scores. This model was adjusted for sex and age. Second, we examined associations between mortality and the general neuroticism factor, both neuroticism facets separately, and both facets together. Sex and age were present in all models, and we tested whether the health-related covariates attenuated these associations. Note that the sex- and age-adjusted models are critical because they do not include possible colliders.

Third, we examined associations between the health-related covariates and (a) general neuroticism, the anxious/tense facet, and the worried/vulnerable facet separately and (b) both facets together. This was done to test whether the general neuroticism factor and the facets, and especially the worried/vulnerable facet, were associated with these variables in different directions. That is, we tested whether Richardson and colleagues’ results could be explained by the facet-level mechanism that we proposed.

## Results

The bifactor structure of neuroticism’s items replicated in the new samples (see Table S1 in the Supplemental Material available online). The first set of analyses (see Table S2) revealed that higher general neuroticism and anxious/tense scores were associated with poorer self-rated health; higher worried/vulnerable scores were associated with better self-rated health.

The second set of analyses (see Table 1) revealed that higher general neuroticism was associated with greater all-cause mortality but only in the sex- and age-adjusted model; the anxious/tense facet was not associated with all-cause mortality. Higher scores on the worried/vulnerable facet, on the other hand, were associated with reduced all-cause mortality in the sex- and age-adjusted model (a key result) and in the fully adjusted model. For specific causes of death, we found a similar pattern of results in sex- and age-adjusted models, but adding the other covariates rendered effects nonsignificant. We did not find a significant association in the sex- and age-adjusted model for cancer when both facets were included simultaneously.

**Table 1.** Associations Between Mortality Risk and the General Neuroticism Factor, the Anxious/Tense Facet, and the Worried/Vulnerable Facet From Models Examining the General Neuroticism Factor and the Facets Separately and From Models Examining the Two Facets Simultaneously (*N* = 321,456)

	Examined separately						Examined simultaneously			
Risk factor and model	General neuroticism		Anxious/tense		Worried/vulnerable		Anxious/tense		Worried/vulnerable	
	HR	*p*	HR	*p*	HR	*p*	HR	*p*	HR	*p*
All causes (4,497 deaths)										
Adjusted for age and sex	1.10 [1.07, 1.14]	< .001	0.94 [0.91, 0.98]	.003	0.86 [0.82, 0.89]	< .0001	0.91 [0.96, 1.04]	.905	0.86 [0.82, 0.90]	< .0001
Adjusted for all covariates	0.99 [0.96, 1.02]	.666	0.96 [0.93, 1.00]	.050	0.90 [0.87, 0.95]	< .0001	0.91 [0.96, 1.04]	.985	0.91 [0.87, 0.95]	< .0001
Cancer (2,912 deaths)										
Adjusted for age and sex	1.04 [1.00, 1.08]	.064	0.92 [0.87, 0.96]	< .0001	0.88 [0.84, 0.93]	< .0001	0.95 [0.90, 1.00]	.068	0.91 [0.85, 0.96]	.001
Adjusted for all covariates	0.96 [0.92, 1.00]	.060	0.94 [0.89, 0.98]	.008	0.93 [0.88, 0.98]	.004	0.96 [0.91, 1.01]	.115	0.94 [0.89, 1.00]	.052
Cardiovascular disease (925 deaths)										
Adjusted for age and sex	1.13 [1.06, 1.22]	.001	0.91 [0.84, 1.00]	.041	0.80 [0.73, 0.88]	< .0001	1.00 [0.90, 1.10]	.923	0.81 [0.72, 0.89]	< .0001
Adjusted for all covariates	0.95 [0.88, 1.02]	.166	0.94 [0.87, 1.02]	.164	0.88 [0.81, 0.97]	.010	0.99 [0.90, 1.10]	.725	0.89 [0.80, 0.99]	.029
Respiratory disease (688 deaths)										
Adjusted for age and sex	1.18 [1.09, 1.28]	< .0001	0.95 [0.86, 1.05]	.289	0.81 [0.73, 0.90]	< .0001	1.04 [0.93, 1.15]	.527	0.80 [0.71, 0.90]	< .0001
Adjusted for all covariates	0.97 [0.89, 1.05]	.470	0.93 [0.85, 1.03]	.154	0.88 [0.79, 0.98]	.021	0.98 [0.88, 1.08]	.641	0.89 [0.79, 1.00]	.060
External causes (422 deaths)										
Adjusted for age and sex	1.25 [1.14, 1.39]	< .0001	1.01 [0.89, 1.14]	.888	0.93 [0.81, 1.07]	.308	1.04 [0.91, 1.20]	.516	0.91 [0.78, 1.06]	.231
Adjusted for all covariates	1.11 [1.00, 1.23]	.049	1.00 [0.89, 1.13]	.988	0.98 [0.85 1.12]	.753	1.01 [0.88, 1.15]	.893	0.97 0[.84, 1.13]	.733

Note: Estimates represent hazard ratios (HRs); 95% confidence intervals are given in brackets. Estimates were first adjusted for age and sex and then, in addition, for other covariates at baseline: health behaviors (smoking status, frequency of alcohol intake, number of types of exercise engaged in, and daily consumption of fruits and vegetables), physical attributes (body mass index, forced expiratory volume in 1 s, systolic blood pressure, and grip strength), reaction time, existing illness (diagnosis of vascular or heart problems, diabetes, cancer, asthma, chronic lung disease, deep vein thrombosis, or pulmonary embolism at baseline), and socioeconomic position (Townsend index score and highest educational qualification). Alpha was set to .001.

The third set of analyses (see Table S3) revealed that for nearly every covariate, higher general neuroticism scores were associated with higher risk, and higher worried/vulnerable scores were protective (another key result).

## Discussion

The worried/vulnerable facet of neuroticism is linked to better health in models that do not include possible colliders. This finding suggests that in addition to considering collider bias as an explanation for why the inclusion of self-rated health causes neuroticism to become protective, one must consider the possibility that self-rated health is a negative suppressor, which reveals the action of a neuroticism facet related to worry and vulnerability. The latter explanation has the same number of parameters as the collider-bias explanation but describes a scenario in which neuroticism has a direct positive effect on health and an indirect negative effect on health via self-rated health.

However, the latter explanation, which we favor, appears to be more consistent with findings from the literature at the time of the study and those that emerged since then. These findings include those relating to the association between self-rated health and mortality (Benyamini & Idler, 1999; Heistaro, Jousilahti, Lahelma, Vartiainen, & Puska, 2001; Idler & Benyamini, 1997), which we cited earlier, and those from the study by Ploubidis and Grundy (2009), who modeled the association between neuroticism and mortality. Their study found that, in women but not men, the direct effect of neuroticism was related to reduced risk and that the indirect effect of neuroticism via a somatic health factor (it loaded on self-rated health) was related to increased mortality. Similarly, another study found that “body vigilance” acted as a suppressor: When it was included in a model, the effect of neuroticism was protective (Weston & Jackson, 2018). Our findings are also consistent with the fact that not all facets of a personality domain are responsible for personality–health associations (e.g., Terracciano et al., 2009) and that although Type 2 diabetes is related to lower neuroticism net of depression (Čukić & Weiss, 2014), Type 1 diabetes risk, which cannot be reduced by vigilance, is related to higher neuroticism, regardless of whether depression is included in the model (Čukić & Weiss, 2016). Finally, a Mendelian randomization analysis by Nagel, Watanabe, Stringer, Posthuma, and van der Sluis (2018) found that a similar facet, which they labeled “worry” was related to lower waist circumference and lower body mass index, both mortality risk factors. Thus, multiple strands point to a health-protective role for neuroticism via vigilance or for one or more neuroticism facets related to vigilance. That said, because Nagel et al. did not use a bifactor analysis to obtain this facet, it is not possible to know whether the relationships that they report reflect associations with the facet or with the general neuroticism factor (see our earlier discussion on this point).

Richardson and colleagues are correct: One should be alert to the possibility of collider bias, and we agree that that was a possible—but not the only—interpretation of our original results. Future studies on the causal association between self-rated health and mortality will be key to understanding which explanation is likely to be correct. Nonetheless, our results as a whole, and the literature, appear to better support an alternative possibility that some neuroticism facets are associated with higher health risks, some are neutral, and some are protective.

## Supplemental Material

WeissOpenPracticesDisclosure – Supplemental material for Conditioning on a Collider May or May Not Explain the Relationship Between Lower Neuroticism and Premature Mortality in the Study by Gale et al. (2017): A Reply to Richardson, Davey Smith, and Munafó (2019) Click here for additional data file. Supplemental material, WeissOpenPracticesDisclosure for Conditioning on a Collider May or May Not Explain the Relationship Between Lower Neuroticism and Premature Mortality in the Study by Gale et al. (2017): A Reply to Richardson, Davey Smith, and Munafó (2019) by Alexander Weiss, Catharine R. Gale, Iva Čukić, G. David Batty, Andrew M. McIntosh and Ian J. Deary in Psychological Science

## Supplemental Material

WeissSupplementalMaterial – Supplemental material for Conditioning on a Collider May or May Not Explain the Relationship Between Lower Neuroticism and Premature Mortality in the Study by Gale et al. (2017): A Reply to Richardson, Davey Smith, and Munafó (2019) Click here for additional data file. Supplemental material, WeissSupplementalMaterial for Conditioning on a Collider May or May Not Explain the Relationship Between Lower Neuroticism and Premature Mortality in the Study by Gale et al. (2017): A Reply to Richardson, Davey Smith, and Munafó (2019) by Alexander Weiss, Catharine R. Gale, Iva Čukić, G. David Batty, Andrew M. McIntosh and Ian J. Deary in Psychological Science
