# Supplementary material for: Conditioning on a Collider May or May Not Explain the Relationship Between Lower Neuroticism and Premature Mortality in the Study by Gale et al. (2017): A Reply to Richardson, Davey Smith, and Munafò (2019)
Source: Psychol Sci. 2019 Feb 22;30(4):633–8. doi: 10.1177/0956797619833325 (PMC6472143; doi:10.1177/0956797619833325)
Supplement: WeissSupplementalMaterial – Supplemental material for Conditioning on a Collider May or May Not Explain the Relationship Between Lower Neuroticism and Premature Mortality in the Study by Gale et al. (2017): A Reply to Richardson, Davey Smith, and Munafó (2019) [file WeissSupplementalMaterial.pdf]

Table S1

Bi-factor structure of neuroticism items in UK Biobank (UKBB), Generation Scotland (GS), and in the data used to develop the Revised Eysenck Personality Questionnaire (EPQ-R)

	General neuroticism			Anxious/tense			Worried/vulnerable		
	UKBB	GS	EPQ-R	UKBB	GS	EPQ-R	UKBB	GS	EPQ-R
Does your mood often go up and down?	**.736**	**.629**	**.635**	-.130	-.224	-.158	-.047	-.018	-.042
Do you ever feel 'just miserable' for no reason?	**.673**	**.574**	**.579**	-.154	-.230	-.169	.027	.057	.034
Are you an irritable person?	**.492**	**.409**	**.491**	.027	-.061	.029	-.043	-.022	-.050
Are your feelings easily hurt?	**.458**	**.437**	**.425**	-.024	.000	-.016	**.399**	**.365**	**.303**
Do you often feel 'fed-up'?	**.708**	**.650**	**.681**	-.158	**-.311**	-.175	-.018	-.016	-.052
Would you call yourself a nervous person?	**.463**	**.590**	**.483**	**.608**	**.438**	**.560**	.026	.048	.000
Are you a worrier?	**.481**	**.508**	**.530**	.161	.089	.151	**.309**	.295	**.396**
Would you call yourself tense or 'highly-strung'?	**.481**	**.578**	**.467**	**.352**	**.311**	**.368**	-.020	-.025	.012
Do you worry too long after an embarrassing experience?	**.403**	**.415**	**.400**	.009	.002	-.006	**.568**	**.614**	**.459**
Do you suffer from 'nerves'?	**.439**	**.615**	**.501**	**.490**	**.357**	**.532**	-.028	-.045	-.007
Do you often feel lonely?	**.452**	**.496**	**.491**	-.027	-.156	.010	.057	.016	-.004
Are you often troubled about feelings of guilt?	**.450**	**.501**	**.446**	-.014	-.044	-.033	**.315**	**.336**	.251
Factor determinacies	.919	.920	.907	.790	.748	.781	.721	.729	.658

Note. Tucker's congruence coefficients for the general factor were .99 for the comparison of the UK Biobank (UKBB) and the Generation Scotland (GS), 1.00 for the comparison of the UK Biobank and the Revised Eysenck Personality Questionnaire (EPQ-R) structure, and .99 for the comparison between the GS and EPQ-R structure. For the anxious-tense facet these congruence coefficients were .93, 1.00, and .93, respectively. For the worried-vulnerable facet these values were .99, .98, and .97 respectively. Root mean square error of approximation estimates for the UKBB, GS, and EPQ-R structures were .048 (90% confidence interval [CI] = .047 to .048), .034 (90% CI = .031 to .037), and .039 (90% CI = .031 to .048), respectively. The comparative fit indices for these structures were .975, .987, and .981, respectively. The Tucker Lewis indices for these structures were .950, .974, and .962, respectively. The standardized root mean square residuals for these structures were .019, .014, and .019, respectively.

Table S2

Multinomial regression of the general neuroticism factor and the two neuroticism facets, onto self-rated health strata.

Self-rated health strata		*RRR*	*SE*	*z*	*p*	*l*-95%	*u*-95%
Good							
	Sex	1.130	.011	12.47	< .001	1.109	1.152
	Age	1.018	.001	29.50	< .001	1.016	1.019
	General neuroticism	1.531	.009	69.85	< .001	1.513	1.549
	Anxious/tense	1.063	.008	8.08	< .001	1.047	1.078
	Worried/vulnerable	0.979	.007	-2.87	.004	0.964	0.993
	Constant	1.250	.042	6.70	< .001	1.171	1.335
Fair							
	Sex	1.577	.019	37.02	<.001	1.539	1.616
	Age	1.025	.001	33.47	< .001	1.024	1.027
	General neuroticism	2.519	.018	128.63	<.001	2.484	2.555
	Anxious/tense	1.080	.009	8.90	<.001	1.062	1.099
	Worried/vulnerable	0.883	.008	-13.15	< .001	0.867	0.900
	Constant	0.203	.009	-37.34	< .001	0.187	0.221
Poor							
	Sex	1.818	.040	27.30	< .001	1.741	1.898
	Age	1.025	.001	18.27	< .001	1.022	1.027
	General neuroticism	3.940	.049	109.25	< .001	3.844	4.038
	Anxious/tense	1.120	.015	8.26	< .001	1.090	1.151
	Worried/vulnerable	0.743	.013	-17.21	< .001	0.719	0.769
	Constant	0.028	.002	-46.38	< .001	0.024	0.032

Note. Reference category is Excellent self-rated health. *RRR* = relative risk ratio, *SE* = standard error, *l*-95% = lower bound of the 95% confidence interval, *u*-95% = upper bound of the 95% confidence interval. Alpha set to .001.

Table S3

Associations between participant characteristics and general neuroticism factor and the anxious/tense and worried/vulnerable facets examined separately and simultaneously

		Examined separately			Examined simultaneously	
		General neuroticism	Anxious/tense	Worried/vulnerable	Anxious/tense	Worried/vulnerable
Current smoker	*OR*	1.27 [1.26, 1.29]	1.44 [1.41, 1.48]	0.77 [0.76,0.79]	0.93 [0.92, 0.95]	0.80 [0.79, 0.82]
	p	< .0001	< .0001	< .0001	< .0001	< .0001
Eats 5+ portions of fruit/vegetables daily	*OR*	0.90 [0.89, 0.91]	1.00 [0.99, 1.01]	1.00 [0.99, 1.02]	0.99 [0.98, 1.00]	1.01 [1.00, 1.02]
	p	< .001	.880	.357	.123	.134
Drinks alcohol daily or almost daily	*OR*	0.98 [0.97, 0.99]	1.02 [1.01,1.03]	1.08 [1.07, 1.09]	0.99 [0.98, 1.00]	1.09 [1.07, 1.10]
	p	< .001	< .0001	< .0001	.075	< .0001
Vascular/heart problems	*OR*	1.22 [1.21, 1.23]	1.05 [1.04, 1.06]	0.99 [0.98, 1.00]	1.07 [1.06, 1.08]	0.96 [0.94, 0.97]
	p	< .001	< .0001	.029	< .0001	< .0001
Diabetes	*OR*	1.21 [1.19, 1.23)	0.82 0.81,0 .84]	0.81 [0.79, 0.83]	0.88 [0.85,0.90]	0.86 [0.84, 0.88]
	p	< .001	< .0001	< .0001	< .0001	< .0001
Asthma	*OR*	1.14 [1.13, 1.15]	1.02 [1.01, 1.04]	1.02 [1.01, 1.04]	1.02 [1.00, 1.04]	1.01 [1.00, 1.03]
	p	< .001	.001	.005	.014	.143
Chronic lung disease	*OR*	1.54 (1.49, 1.59]	1.04 [.99, 1.08)	0.91 [0.87, 0.96]	1.09 [1.04, 1.14)	0.97 [0.83, 0.92]
	p	< .001	.097	< .0001	< .0001	< .0001
Cancer	*OR*	1.02 [1.01, 1.04)	0.99 [0.97, 1.01]	0.99 [0.97, 1.01]	0.99 [0.97, 1.01]	1.00 [0.98, 1.02]
	p	.001	.170	.454	.243	.858
Deep vein thrombosis	*OR*	1.13 [1.10, 1.16]	0.91 [0.88,0 .94]	0.92 [0.88, 0.95]	0.93 [0.89, 0.96]	0.95 [0.91, 0.99]
	p	< .001	< .0001	< .0001	< .0001	.010
Pulmonary embolism	*OR*	1.14 [1.09, 1.19]	0.89 [0.84, 0.94]	0.92 [0.87, 0.98]	0.90 [0.84, 0 .95]	0.97 [0.91, 1.03]
	p	< .001	< .0001	.006	< .0001	.330

Has a degree	*OR*	0.80 [0.79, 0.81]	1.05 [1.04, 1.05]	1.12 [1.11, 1.14]	1.00 [0.99, 1.01]	1.12 [1.11, 1.14]
	p	< .001	< .0001	< .0001	.810	< .0001
Exercise taken	*b*	-0.148 [-0.152,0 .143]	-0.004 [-0.005, 0.005]	0.043 [0.042, 0.053]	-0.023 [-0.028, -0.017]	0.058 [0.052, 0.064]
	p	< .0001	.880	< .0001	< .0001	< .0001
BMI (kg/m^2)	*b*	0.261 [0.243, 0.279]	-0.737 [-0.757, -0.712]	-0.560 [-0.582, -0.537]	-0.640 [-0.626, -0.618]	-0.252 [-0.276 -0.227]
	p	< .0001	< .0001	< .0001	< .0001	< .0001
SBP (mm Hg)	*b*	-0.421 [-0.487, -0.355]	0.422 [0.346, 0.498]	-0.500 [-0.584, -0.416]	0.757 [0.673, 0.841]	-0.864 [-0.958, -0.771]
	p	< .0001	< .0001	< .0001	< .0001	< .0001
Grip strength (kg)	*b*	-0.761 [-0.789, -0.734]	-0.411 [-0.443, -0.379]	-0.248 [-0.283, -0.213]	-0.387 [-0.422, -0.352]	-0.062 [-0.101, -0.023]
	p	< .0001	< .0001	< .0001	< .0001	.002
FEV1 (l)	*b*	-0.032 [-0.034, 0.030]	-0.002 [-0.004,0 .001]	0.023 [0.020, 0.026]	-0.013 [-0.016, 0.016]	0.030 [0.027, 0.033)
	p	< .0001	.156	< .0001	< .0001	< .0001
Reaction time (ms)	*b*	4.72 [4.31, 5.13]	5.07 [4.59, 5.54]	0.937 [0.412, 1.47]	5.78 [5.26, 6.30]	-1.84 [-2.43, -1.26]
	p	< .0001	< .0001	< .0001	< .0001	< .0001
Townsend index	*b*	0.242 [0.231, 0.254]	-0.048 [-0.061, -0.035]	-0.178 [-0.192, -0.164]	0.026 [0.012, 0.040]	-0.191 [-0.207, -0.164]
	p	< .0001	< .0001	< .0001	< .0001	< .0001

Note. Estimates are per standard deviation of general neuroticism factor or the facets; estimates are odds ratios (*OR*s) or regression coefficients (*b*). 95% confidence intervals are in brackets and have been adjusted for age and sex. Estimates are presented first from models examining the general neuroticism factor and the anxious/tense and worried/vulnerable facets separately, and then from models examining the facets simultaneously. Disease categories are based on physician diagnoses. BMI = body mass index, SBP = systolic blood pressure, FEV1 = forced expiratory volume in one second. Townsend index is the measure of social deprivation. Although Richardson, Davey Smith, and Munafò (2018) examined grip strength for each hand separately, we did not do so in our original report (Gale et al., 2017) and so prefer not to do so here. Alpha set to .001.

References

Gale, C. R., Čukić, I., Batty, G. D., McIntosh, A. M., Weiss, A., & Deary, I. J. (2017). When is higher neuroticism protective against death? Findings from UK Biobank. *Psychological Science, 28*, 1345-1357. doi:10.1177/0956797617709813

Richardson, T. G., Davey Smith, G., & Munafò, M. R. (2018). Conditioning on a collider may induce spurious associations: Do the results of Gale et al. (2017) support a health-protective effect of neuroticism in population sub-groups? *Psychological Science*.
